# OP56 Hospital-Based Health Technology Assessment: Barriers And Facilitators In France, Hungary, Italy, Kazakhstan, Poland, Switzerland, And Ukraine

**DOI:** 10.1017/S0266462324001181

**Published:** 2025-01-07

**Authors:** Rossella Di Bidino, Iga Lipska, Monika Kukla, Bertalan Nemeth, Rok Hren

## Abstract

**Introduction:**

The adoption and development of health technology assessment (HTA) in the hospital setting (HB-HTA) differs among countries, but in all cases, it encounters barriers and facilitators. An analysis was conducted to promote international cooperation to develop strategies both to enforce common facilitators and overcome common barriers. HTA specialists from seven countries (at least two per country) contribute to the study.

**Methods:**

HTA experts from countries in Western Europe (three), Central and Eastern Europe (three), and Central Asia (one) voluntarily participated in the project. They provided a description of the scenario that HB-HTA faces in their country, then a two-round Delphi study was conducted. The survey was based on twelve statements that were categorized into external and internal barriers and external and internal facilitators. Next, panel experts ranked statements on a seven-point Likert scale with a median agreement score greater than or equal to six and an interquartile range (IQR) greater than or equal to one accepted as reaching consensus. The goal was to identify similarities and differences in the HB-HTA scenarios among countries.

**Results:**

Fifteen experts from France, Hungary, Italy, Kazakhstan, Poland, Switzerland, and Ukraine contributed to the analysis. Among the twelve statements, six were ranked as reaching consensus (two barriers, four facilitators). One external and one internal barrier, which reached consensus, were (i) lack of formal recognition of the role of HB-HTA in national/regional legislations and (ii) limited human resources. Two external facilitators were (iii) creation of a network among hospitals performing HB-HTA and (iv) dissemination of HB-HTA methods and activities, while two internal facilitators were (v) top hospital management support in evidence-based decision-making and (vi) training initiatives dedicated to HB-HTA.

**Conclusions:**

The analysis showed a consensus on the barriers and facilitators for HB-HTA. This creates the opportunity for internationally developed strategies to enforce facilitators and support the adoption and sustainability of HB-HTA. Future research should extend the analysis to other countries and include the results of the HB-HTA survey conducted in 2023 by the HB-HTA Interest Group.

